# Practical Hints upon Medical Examination for Life Assurance.—III

**Published:** 1908-08-22

**Authors:** William P. S. Branson

**Affiliations:** Junior Medical Officer, Sun Life Office.


### Life Assurance.

### PRACTICAL HINTS UPON MEDICAL EXAMINATION FOR LIFE ASSURANCE—III.

By WILLIAM P. S. BRANSON, M.D., M.R.C.P., Junior Medical Officer, Sun Life Office.

Heart-murmurs (*continued*).

Having dealt with the apical systolic murmur which leaves no doubt that it is due to organic valvular disease, we pass to apical systolic murmurs of more dubious significance. Of these, the following may be distinguished:—

1. A short rough systolic murmur heard above and within the point of impulse. It has a character suggestive of nearness to the ear, and is probably to be considered exocardial, the expression, perhaps, of a milk-spot upon the pericardium. In the absence of evidence of cardiac hypertrophy it may be ignored.

2. A blowing systolic murmur of maximum intensity during inspiration. This is the commonest murmur of the group under notice. It is not to be distinguished from an organic murmur except by its variability, but the latter is a notable characteristic, for during expiration it becomes exceedingly faint and may disappear entirely. It is met with most often when the heart is beating somewhat tumultuously under the influence of emotion. The items for its identification are therefore these: Increase in loudness during inspiration, decrease during expiration, and disappearance when the chest is emptied by a forced expiration. If to these be added an agitated beating of the heart and no sign of cardiac hypertrophy, the diagnosis is tolerably certain, and the condition, as regards longevity, of no moment.

3. A roughening of the first sound rendering it murmurous, yet not to be fairly described as a murmur. This, like the last, is common in emotional young adults, and is fairly often associated with a poor physique and postural albuminuria, but not with hypertrophy. In some cases a definite murmur appears when the proposer assumes a recumbent position; in others no alteration ensues. As a rule there is nothing in the past history to suggest the likelihood of organic valvular disease, yet the condition is not variable, but constant. It is open to question whether or not this sound should be considered as evidence of disease, and therefore the examiner should state the pros and cons in as detailed a manner as possible.

Murmurs heard at the aortic base, both systolic and diastolic, whether typical or not of aortic disease are so suggestive of danger as to exclude the life concerned. A systolic murmur at the pulmonary base, in the absence of a thrill and of all other evidences of cardio-vascular disturbance, is negligible. There is, however, a short double murmur occasionally to be heard over the pulmonary region of the base of the heart which has the peculiar superficial quality suggestive of an exocardial origin. Yet it is known that aortic murmurs are sometimes better heard over the pulmonary than over the aortic base, and therefore the greatest caution is necessary in assigning the murmur indicated to an exocardial source.

It will not be overlooked that the diastolic murmur of aortic regurgitation is often better heard down the left border of the sternum than at the aortic base. Of the other murmurs which reach their maximum intensity at some point behind the sternum other than the base there is a rough one, systolic in time, and mid-sternal in place, though often to be heard widely over the præcordial region. This murmur represents a developmental anomaly, most often a deficiency in the "undefended space" of the interventricular septum. The condition is compatible with perfect health and activity, yet it cannot be ignored in life assurance, for it is known that deformed hearts are more liable than others to endocarditis. Other things being favourable and the heart not hypertrophied, the case may rank as a mild mitral regurgitation.

There may be heard occasionally, especially at the beginning of an examination, a systolic humming sound situated behind the upper and mid-sternum. This sound has the characters of a venous hum and may generally be dissipated by a few deep inspirations. It is of no significance. There is yet another harsh systolic murmur, at its maximum during inspiration, heard over the upper lobes of the lungs below the middle and outer parts of the clavicle. This murmur is extremely variable and of no importance.

Irregularity in the rhythm of the heart occurring apart from other evidences of disease is a common event. In young lives—prior, that is to say, to the age at which the onset of degenerative processes may be suspected, the condition may be due to an excessive consumption of tobacco. But there may be no such simple explanation. Yet the symptom has no serious significance at this age unless it be excessive. At a later age the problem of an irregular heart is by no means easy of solution. Myocardial affections of the gravest kind may for a time be represented by no other evidence than this; while, on the other hand, the hearts of elderly people may stumble occasionally, over a long period of years, without untoward results. The family history as regards longevity, the quality of the heart-sounds, the condition of the urine, and the general state of preservation of the proposer are all items to be given weight.

Alterations in Size.

Hypertrophy is the only alteration in size which can be identified. It will not be forgotten that the level of the apex beat is largely dependent upon the conformation of the chest, being lower in the long chest than in the broad one. If, then, the impulse be in the sixth space, it is of service that the examiner should state whether the shape of the chest is such as renders the position normal, for otherwise the reader of the report is left in doubt whether or not the case is to be treated as one of cardiac hypertrophy. If the hypertrophy be undoubted, its explanation and extent require to be fully detailed, unless the cause lies in lesions so gross as to preclude all possibility of assurance. A slight approximation of the impulse to the line of the nipple is so common an event in the case of people past middle age as to come within the limits of normal variation, provided that the case is otherwise free from suspicion. Prolonged application to violent athletic exercises, particularly running, rowing, and long-distance cycling, may produce a similar result in young lives without entailing evil omen. It is important, therefore, that evidences of cardiac hypertrophy should not be baldly given without comment, lest an average life be credited unduly with adherent pericardium or renal disease or arteriosclerosis.

